# Identification and Characterization of SRSF2 as a Splicing-Relevant Factor Associated with the Distribution of Membranous to Secreted PD-L1, Exemplarily Considered on Human Renal Tissue, Including Renal Cell Carcinoma

**DOI:** 10.3390/cancers17213463

**Published:** 2025-10-28

**Authors:** Tim Hohmann, Urszula Hohmann, Faramarz Dehghani, Hendrik Borgmann, Olaf Grisk, Galyna Pryymachuk, Simon Jasinski-Bergner

**Affiliations:** 1 Department of Anatomy and Cell Biology, Medical Faculty, Martin Luther University Halle-Wittenberg, Grosse Steinstrasse 52, 06108 Halle (Saale), Germany; tim.hohmann@uk-halle.de (T.H.); urszula.hohmann@uk-halle.de (U.H.); faramarz.dehghani@medizin.uni-halle.de (F.D.); 2 Urology and Children Urology Clinic, Brandenburg Medical School Theodor Fontane, Hochstrasse 29, 14770 Brandenburg, Germany; hendrik.borgmann@uk-brandenburg.de; 3 Institute of Physiology, Brandenburg Medical School (MHB), Theodor Fontane, 14770 Brandenburg an der Havel, Germany; olaf.grisk@mhb-fontane.de; 4 Institute of Anatomy, Brandenburg Medical School (MHB), Theodor Fontane, 14770 Brandenburg an der Havel, Germany; galyna.pryymachuk@mhb-fontane.de

**Keywords:** cancer, tumor immunology, immune checkpoint, PD-L1, B7-H1, alternative splicing, therapeutic antibody

## Abstract

**Simple Summary:**

Monoclonal antibodies have revolutionized anti-tumoral therapies. They have improved response rates and overall survival times in treated patients in the majority of tumor entities. Of these monoclonal antibodies, those targeting tumor-immune checkpoint axes and thereby inhibiting immunomodulatory interactions between tumor cells and immune effector cells are of particular relevance. The interaction of the ligand PD-L1 on tumor cells with the inactivating PD-1 receptor on immune effector cells, such as T killer cells, is of immense relevance. Monoclonal antibody therapies are very expensive, so the gene expression of targeted molecules, such as PD-L1, must be characterized for improved efficacy. During malignant transformation, alternative splicing in tumor cells is also altered. PD-L1 is highly subject to alternative splicing. This study identified SRSF2, a splicing factor whose protein levels are associated with the distribution of membranous to secreted PD-L1 splice variants, and further investigated SRSF2 gene expression and its tumor biological relevance.

**Abstract:**

**Background:** The immunomodulatory molecule PD-L1 and its immunological tolerance-mediating interaction with the PD-1 receptor on many immune effector cells represent one of the most important tumor immune checkpoint axes in antibody-based anti-tumor therapies. Furthermore, PD-L1 is subject to alternative splicing, whereby, in addition to the membrane-bound PD-L1, secreted PD-L1 is also formed as an additional splice variant. This also exerts its effects in the tumor microenvironment, even away from the actual tumor cells, and contributes additional benefits to immune evasion of the tumor. **Methods:** To examine the association of the splicing factor SRSF2 with the PD-L1 splicing pattern, respective SRSF2 overexpression and knockdown experiments were performed. The precise characterization of SRSF2 followed in human kidney tissue samples and RCCs, including immunofluorescence staining. The impact of the known oncogenic SRSF2 on the host cell transcriptome was further analyzed by RNA sequencing analyses in SRSF2 overexpression and knockdown experiments. **Results:** In this original research article, the trans splicing factor SRSF2 is identified to be associated with the shift in the alternative splicing pattern of PD-L1 towards the secreted splice variant. The impact of SRSF2 on the cellular transcriptome was demonstrated, and its involvement in the process of malignant transformation, which is obviously also directly linked to immune evasion. **Discussion:** The optimization of anti-tumor therapies based on monoclonal antibodies against immunomodulatory axes such as PD-1 and PD-L1, including necessary cost reductions, requires the detailed characterization of the gene expression and gene regulation of such molecules, as well as comprehensive molecular biological diagnostics of the tumor sample before putative therapy formulations, e.g., antibody panel collection. **Conclusion:** Thus, both the amount of PD-L1 protein produced and its splicing pattern are crucial for therapy success and for selecting the most effective therapeutic antibodies.

## 1. Introduction

Structurally, the B7 protein group belongs to the immunoglobulin superfamily (IgSF), a very extensive protein family that includes not only the immunoglobulins (including membranous B cell receptors) themselves but also other protein families with so-called Ig-like domains. This includes, among others, T cell receptors and HLA class I and II molecules, cell adhesion molecules such as ICAM, VCAM, and NCAM, and the B7 protein family. The name derives from the presence of one or more extracellular immunoglobulin (Ig) or Ig-like domains (approximately 70–110 amino acids) with an antiparallel β-sheet sandwich barrel as a structural feature. Meanwhile, multiple variants of topology and structure have been identified, with four main variant types, named V-set (IgV), C1-set (IgC1), C2-set (IgC2), and I-set (IgI) [1]. In fact, members of the B7 family belong to the type I IgSF. They are single-stranded membrane proteins whose structural design enables reversible protein–protein interactions on the cell surface, e.g., with immune effector cells [2].

These genes emerged with the evolution of jawed vertebrates, coinciding precisely with the development of the adaptive immune system [2]. Indeed, the proteins of the B7 family are immunomodulatory molecules that, depending on the respective ligand–receptor interaction, can act in either a costimulatory or coinhibitory fashion [3]. B7 molecules play a crucial role in immune modulation, and some of these receptor–ligand interactions have now been characterized as (tumor-) immune checkpoint (ICP) axes with enormous therapeutic relevance in a variety of tumor entities. PD-L1, which acts as a ligand for the inhibitory PD-1 receptor, stands out in particular. The PD-1 receptor is expressed on various immune effector cells, including monocytes, dendritic cells, macrophages, B cells, T cells, and NK cells [4].

Although PD-L2 can also bind to PD-1 as a ligand with significantly higher affinity than PD-L1 [5], the interaction of PD-1 with PD-L1 plays the decisive role in antibody-based anti-tumor immunotherapies [4]. The reason for this lies in PD-L1 gene expression, which is strongly enhanced upon malignant transformation. Physiologically expressed predominantly in immune-privileged tissues, such as the placenta [6], and involved in mediating immunological tolerance, its gene expression is detectable in many solid and hematopoietic tumors after malignant transformation. Here, PD-L1 makes a very strong contribution to immune evasion by causing reduced IL-2 production, cell cycle arrest, and promotion of exhaustion in immune effector cells after binding to its receptor PD-1, for example, on tumor-infiltrating lymphocytes (TILs, e.g., CD8+ cytotoxic T lymphocytes (CTLs)) [7,8].

**Figure 1:**
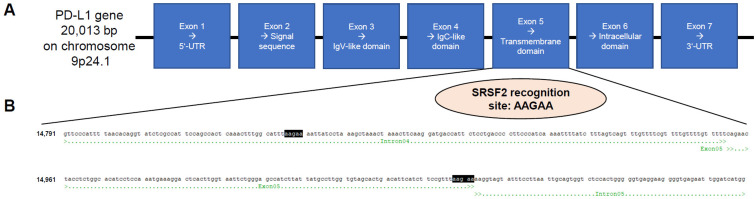
Scheme of the PD-L1-encoding gene with subdivision of exons and introns (**A**) as well as the DNA sequence of exon 5, which encodes for the transmembrane domain with the highlighted binding motif of the trans splice factor SRSF2 at the direct exon/intron border (**B**).

The PD-L1 gene is approximately 20 kbp in size and located on the short arm of chromosome 9 at the position 9p24.1. The primary transcript contains seven exons. The IgV-like domain is encoded in exon 3 and the transmembrane domain in exon 5 (Figure 1A). Indeed, the PD-L1 primary transcript succumbs to alternative splicing. To date, six different splice variants have been described in the literature [4]. In addition to the membranous full-length PD-L1 variant, there is another truncated membranous splice variant without exon 3 reported in the literature (delta IgV-like domain (exon 3)) and a total of four different splice variants without a transmembrane domain (exon 5), whose corresponding proteins are secreted and no longer membrane-bound: PD-L1-1, PD-L1-3, PD-L1-9, and PD-L1-12. It has already been shown that this altered splicing pattern shifting towards secreted splice variants correlates with disease progression [9,10,11].

Considering the therapeutic use of monoclonal antibodies (mAbs) against PD-L1 in malignant tumor treatment such as atezolizumab, avelumab, and durvalumab, it is already known that secreted PD-L1 can effectively block immune effector cells in the tumor microenvironment. Since secreted PD-L1 is also bound by the therapeutic antibodies, these antibodies cannot contribute to the induction of antibody-dependent cellular phagocytosis (ADCP), anti-body-dependent cellular cytotoxicity (ADCC), and complement activation of the PD-L1-expressing tumor cell, as would be the case with membrane-bound PD-L1, in addi-tion to the blockade of the ICP axis per se. Therefore, tumor cells with a higher expression of secreted PD-L1 splice variants respond worse to the respective antibody therapies [4].

Renal cell carcinomas (RCCs) are the most common type of cancer that arises in the kidney and are associated with the following risk factors, among others: smoking, obesity, high blood pressure, and exposure to cancerogenic chemicals such as trichloroethylene [12]. This tumor entity has so far shown very poor response rates to anti-tumor therapies, with mAb-based therapies targeting ICP molecules providing the hoped-for breakthrough. PD-L1, in particular, is representative of ICP molecules. A meta-analysis of 1644 patients in eight studies showed that PD-L1 expression significantly correlates with both overall survival and disease-free survival [13].

In this article, serine/arginine-rich splicing factor 2 (SRSF2) is identified as an associated splicing factor with great relevance for the distribution of membranous to secreted PD-L1 protein. Both overexpression and downregulation of this splice factor lead to an altered ratio of membrane-bound to secreted PD-L1. The colocalization of SRSF2 and PD-L1 in various human tissues was also demonstrated. Furthermore, the precise significance of the splice factor SRSF2 for the survival of tumor patients with or without therapeutic PD-L1 antibody treatment was characterized. The impact of SRSF2 expression was further analyzed by RNA-Seq-based transcriptome analyses of respective SRSF2-overexpressing and knockdown transfectants with regard to which other tumor immunology- or tumor biology-relevant genes were influenced by SRSF2.

## 2. Materials and Methods

### 2.1. Data Sets

The data bank analysis was based on the TCGA-KIRC, TCGA-KICH and TCGA-KIRP RNAseq data [14,15,16]. These sets contain sequencing data of 1028 patients, including 91 chromophobe RCCs, 323 papillary RCCs, 600 clear cell RCCs, and 14 differently classified cases. In 18 cases, treatment was applied prior to sequencing and thus the cases were excluded, as this could alter the results. For 2 patients with a chromophobe and 1 patient with papillary RCC, no follow-up or survival time data was available. Thus, in sum, 35 cases were excluded from analysis, leaving 89 chromophobe RCCs, 322 papillary RCCs, and 582 clear cell RCCs for analysis.

### 2.2. Data Analysis

Differential gene expression was performed using DESeq2, implemented in MATLAB R2021a (https://github.com/jbmorlot/DESeq2-matlab?tab=readme-ov-file, accessed on 12 June 2023) [17], as described previously [18,19]. For false discovery rates below 0.05, genes were considered to be differently expressed. MatSurv v1.1.03 (https://de.mathworks.com/matlabcentral/fileexchange/64582-matsurv, accessed on 12 June 2023) was used to analyze and visualize survival times [20]. High- and low-expressing cases were identified by median split, and statistical analysis of survival times was performed using the log-rank (Mantel–Cox) test.

### 2.3. RNA Extraction, cDNA Synthesis, RNA Sequencing Analyses, qPCR, and Generation of the Vectors for Overexpression

The RNA was extracted by usage of the NucleoSpin RNA, Mini Kit (Macherey-Nagel, Düren, Germany). After concentration determination on a NanoDrop instrument (Thermo Fisher, Waltham, MA, USA), cDNA synthesis with the RevertAid H Minus First Strand cDNA Synthesis Kit (Thermo Fisher) followed, or alternatively RNA sequencing analyses at the Core Unit “DNA technologies” (PD Dr. Knut Krohn, University of Leipzig, Medical Faculty, Germany) were performed, as recently described [19].

For mRNA quantification, three biological replicates of each analyzed cell line or transfectant were analyzed on a LineGene 9600 qPCR cycler (Biozym Scientific GmbH, Hessisch Oldendorf, Germany) with GoTaq qPCR MasterMix (Promega, Madison, WI, USA), with GAPDH as a housekeeper gene for determination of relative copy numbers. The following oligonucleotides were employed for qPCR (5′ to 3′): SRSF2fw GTGGACAACCTGACCTACCG, SRSF2rev GTCGACCGAGATCGAGAACG, memPD-L1fw CCATACAGCTGAATTGGTCATCCC, memPD-L1rev GAGGCTCCTTGTTCAGAAGTATCC (slightly modified according to Mahoney et al., 2019 [9], GAPDHfw CAAGGTCATCCATGACAACTTTG (Thermo Fisher), and GAPDHrev GTCCACCACCCTGTTGCTGTAG (Thermo Fisher).

The SRSF2 overexpression vector was cloned by amplification of the SRSF2 coding sequence (CDS) from HEK293T cDNA by PCR in a GeneExplorer 96 Thermocycler (Biozym) and cloned with the restriction enzymes (AgeI and XhoI, both Promega) and T4 DNA ligase (Thermo Fisher) into the pmR-mCherry vector (Takara Bio, Mountain View, CA, USA), replacing the CDS encoding for the mCherry protein by usage of the following oligonucleotides (5′ to 3′): clSRSF2fw AAAACCGGTGCCACTCAGAGCTATGAGCTACG and clSRSF2rev: AAACTCGAGCTTAAGAGGACACCGCTCCTTC.

### 2.4. Cell Culture, Cell Transfection, and Flow Cytometry

HEK293T cells were cultured in Dulbecco’s modified Eagle’s medium (DMEM, Thermo Fisher), and JEG-3 cells were maintained in RPMI 1640 medium (Thermo Fisher), supplemented with 10% (*v*/*v*) fetal bovine serum (PAA, Pasching, Austria), 2 mM L-glutamine (Lonza, Basel, Switzerland), and 1% penicillin/streptomycin (*v*/*v*; PAA). All human cell lines used in this study were purchased from the American Type Culture Collection (ATCC, Manassas, VA, USA). Cell transfection with the SRSF2 overexpression vector or, alternatively, with the pmR-mCherry mock control vector was performed with the TurboFect reagent (Thermo Fisher), while the Silencer Select siRNAs against SRSF2 (ID s12730) and the siRNA-negative control #1 (both Thermo Fisher) were transfected with the RiboJuice reagent (Merck, Darmstadt, Germany) according to the instructions of each manufacturer.

The measurement of membranous PD-L1 was performed by flow cytometry on a FACSCelesta system (BD Biosciences, Franklin Lakes, NJ, USA) applying the PE Mouse Anti-Human PD-L1 (clone 29E.2A3; BD) as well as the respective isotype control (BD).

### 2.5. Western Blot, ELISA, Immunochemical Staining, and Immunofluorescence

Total protein was extracted, concentrations were determined with the Pierce BCA Protein Assay Kit (Thermo Fisher), and Western blots were performed with 4 to 12% Bis-Tris precast polyacrylamide gels (NuPAGE, Thermo Fisher). Subsequently, the proteins were transferred to a nitrocellulose membrane (iBlot Transfer stacks; Thermo Fisher). Then, blocking in 5% (*w*/*v*) skim milk powder TBS-T (Carl Roth, Karlsruhe, Germany) followed. Afterwards, the blots were incubated overnight at 4 °C with the respective primary antibodies: polyclonal rabbit anti-SRSF2 antibody (PA5-12402, Thermo Fisher) and the rabbit anti-GAPDH mAb (14C10, Cell Signaling), which served as a housekeeping gene. After the washing steps, the incubation (overnight at 4 °C) with the respective secondary antibody (anti-rabbit HRP-linked antibody (Cell Signaling)) followed.

For signal detection, the Pierce ECL Western blotting substrate (Thermo Fisher) was employed on a VWR Chemi Premium Imager unit (Avantor, Radnor, PA, USA).

The soluble PD-L1 splice variant was quantified in the supernatants of the corresponding transfectants using the Human PD-L1 ELISA Kit (Thermo Fisher) according to the manufacturer’s instructions. The exact same supernatants were used as those from the transfectants (SRSF2 siRNA knockdown or SRSF2 overexpression) that were also analyzed by qPCR and FACS, 72 h after chemical transfection of the cells.

After blocking of endogenous peroxidase activity using a hydrogen peroxide solution (Carl Roth, Karlsruhe, Germany), washing and permeabilization with PBS/Triton occurred (AppliChem, Darmstadt, Germany). Cells were then incubated with normal goat serum (Merck, Darmstadt, Germany) to reduce non-specific binding before the primary antibody (1:100 rabbit anti-SRSF2, Thermo Fisher, Walltham, MA, USA) was applied, followed by a biotinylated secondary antibody (Sigma, Darmstadt, Germany). ExtrAvidin^®^ peroxidase (Sigma) and diaminobenzidine (DAB) (Sigma) were used for signal detection, resulting in a brown precipitate. Finally, cells were counterstained with Mayer’s hematoxylin (Merck), dehydrated in ethanol (Carl Roth), cleared in xylene (Carl Roth), and embedded in Entellan (Merck) for microscopic analysis.

Human kidney specimens (*n* = 12) were embedded in Tissue-Tek^®^ O.C.T. Compound (Sakura Finetek, Alphen aan den Rijn, The Netherlands), rapidly shock-frozen in liquid nitrogen, and stored at −80 °C. Cryosections (20 µm thickness) were prepared at −18 °C, mounted on SuperFrost^®^ Plus slides (Carl Roth), and air-dried for 60 min at 37 °C.

Sections were fixed in 2% paraformaldehyde (PFA) for 5 min at room temperature (RT), rinsed five times for 5 min each in phosphate-buffered saline (PBS), and permeabilized with 0.1% Triton X-100 and 0.05% Tween-20 in PBS for 5 min. Blocking was performed with 5% normal donkey serum (Sigma-Aldrich Chemie GmbH, Eschborn, Germany) containing 0.05% Tween-20 in PBS for 60 min at RT.

Specimens were incubated overnight at 4 °C with primary rabbit anti-SRSF2 antibody (#PA5-12402, Thermo Fisher; Lot AC4653921) diluted 1:70 in Antibody Dilution Buffer (DCS, #AL120R100, Hamburg, Germany).

After three washes in PBS/0.05% Tween-20 (5 min each), sections were incubated for 1 h at RT with Alexa Fluor-conjugated donkey IgG (H + L) secondary antibodies combined with fluorescent wheat germ agglutinin (WGA)–Alexa Fluor 633 conjugate (Thermo Fisher, #W21404), diluted 1:300 and 1:200, respectively, in Antibody Dilution Buffer containing 1 µg/mL DAPI (Thermo Fisher Scientific, D1306). Slides were then washed once in PBS/0.05% Tween-20 (5 min) and twice in PBS before being mounted with ProLong™ Gold Antifade Mountant (Thermo Fisher Scientific, P10144).

Control sections for autofluorescence and non-specific secondary antibody binding were processed identically but without the addition of primary antibodies and WGA.

Confocal images were acquired at RT using sequential scanning mode to avoid spectral overlap (Leica Stellaris 5 DMI8 microscope equipped with Diode 405 and WLL lasers; LAS AF software, v2.6.0, build 7266; Leica Microsystems CMS GmbH, Wetzlar, Germany; objective: HCX PL APO CS 40×/1.25 OIL UV, Leica DMI6000B). Device-specific software was used to adjust signal intensities, generate z-stack images, and create maximum intensity projections (MIPs). For all samples, identical acquisition parameters (laser power, detector gain, and offset settings) were applied to ensure comparability of SRSF2 signal intensities across tissue specimens. Renal tissue images representing the distinct cortical regions were independently reviewed by the authors.

Confocal micrographs of kidneys were analyzed in QuPath [21]. We defined three types of regions of interest (ROI) and manually annotated them for each image: (i) glomeruli only, (ii) tubulo-interstitium (non-glomerular parenchyma), and tubuli only (proximal and distal tubuli). Several ROIs of each type were drawn within a single image, depending on the presence of anatomical structures. Cells were segmented in QuPath, and SRSF2-positive cells were selected using a single fixed intensity threshold applied uniformly to all ROIs. The result for each ROI was the percentage of SRSF2-positive cells (positive/total × 100), exported for further analysis.

### 2.6. Software, Statistics, and Tissue Specimen Collection

All statistically significant (two-tailed Student’s *t*-test; with *p* ≤ 0.05) calculations were performed and visualized in Microsoft Excel 2016 (Microsoft Corporation, Redmond, WA, USA) each time for three biological replicates.

Human kidney tissue samples were obtained during routine nephrectomies at the Department of Urology at the University Hospital Brandenburg an der Havel (UKB). Immediately following organ removal, tissue dissection was performed by an experienced pathologist at the UKB’s Institute of Pathology, obtaining exclusively tumor-free tissue.

## 3. Results

The RNA-binding protein serine/arginine-rich splicing factor 2 (SRSF2) belongs to the SR protein family, which is relevant for mRNA splicing and mRNA stability [22].

During splicing, SRSF2 promotes exon recognition by binding to exonic splicing enhancer (ESE) motifs in precursors of mRNA (pre-mRNA) through its RNA-binding domain, with the SRSF2 recognition site being AAGAA [23]. Due to the aforementioned special relevance of PD-L1 in antibody-based anti-tumor immunotherapies, particularly its presence as a membrane-bound or secreted protein, the PD-L1 sequence was analyzed for the presence of SRSF2 recognition sites. It was found that a corresponding binding site for this splicing factor is located directly at the end of exon 5 (transmembrane domain) and intron 5 (Figure 1B).

**Figure 2:**
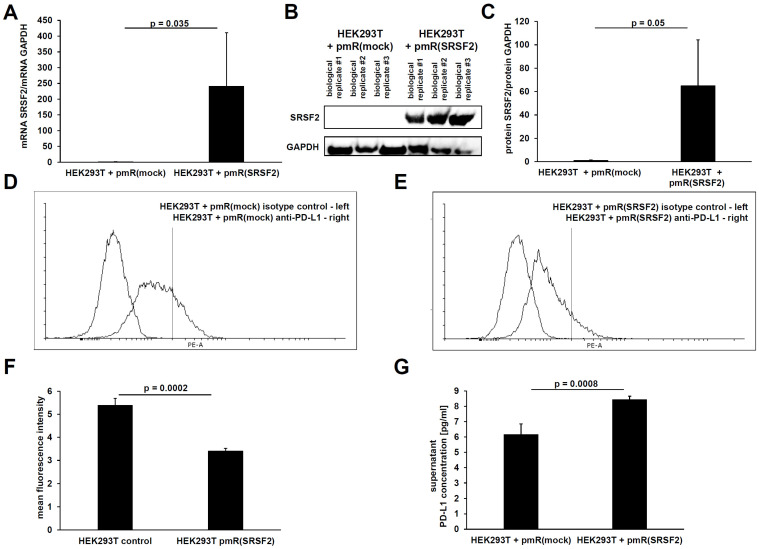
(**A**) qPCR analyses of the relative SRSF2 mRNA level upon transient transfection of HEK293T cells with the SRSF2 expression vector and respective vector control after 72 h and the corresponding SRSF2 protein levels determined by Western blot (**B**) with respective relative quantification to the GAPDH controls by pixel counting (**C**). Membranous PD-L1 protein was quantified by flow cytometry after 72 h of transient transfection with either the vector control or SRSF2 overexpression vector (**D**,**E**). The results of three biological replicates of flow cytometry were visualized as the mean fluorescent intensity (**F**), and the secreted PD-L1 protein was also quantified in the corresponding cell culture supernatants by ELISA (**G**).

To test a possible association for the splicing of the full-length PD-L1 mRNA to the shorter secreted splice variant, an SRSF2 overexpression vector was first cloned. To validate the functionality of this SRSF2 overexpression vector, HEK293T cells were transiently transfected with this SRSF2 overexpression vector and with the vector control. After 72 h, the transfectants were characterized for SRSF2 overexpression at the transcript and protein levels. A statistically significant (*p* = 0.035) overexpression was observed at the transcript level (Figure 2A). It should be noted that after the end of the qPCR (after 40 PCR cycles), all samples were separated on an agarose gel. Only one specific PCR product of the correct size was formed in each case (SRSF2 and GAPDH). It is also noteworthy that the HEK293T + pmR (mock) transfectant is also SRSF2 mRNA-positive.

In the next step, SRSF2 overexpression was quantified at the protein level using Western blot analysis (Figure 2B). This effect was also statistically significant at the protein level (*p* = 0.05), which could be calculated via the cumulative signal intensity (Figure 2C). It should be noted again that the HEK293T + pmR(mock) transfectant is also positive for the SRSF2 protein, but the measurement signal in the overexpressing HEK293T + pmR(SRSF2) transfectant was so strong and bordering on supersaturation that the detection was stopped at this point.

Subsequently, the membrane-bound and secreted PD-L1 protein levels were determined in the two transfectants. Quantification of the membrane-bound PD-L1 protein was performed using flow cytometry. This caused a statistically significant (*p* = 0.0002) decrease in the membrane-bound PD-L1 protein as a direct consequence of overexpression of the splice factor SRSF2. This can be seen in the two representative overlays (Figure 2D,E) and in the statistical analysis using the mean fluorescence intensity calculation (Figure 2F). An ELISA was used to quantify secreted PD-L1 protein in the supernatants of the respective transfectants. This revealed a statistically significant (*p* = 0.0008) increase in secreted PD-L1 protein following SRSF2 overexpression (Figure 2G).

**Figure 3:**
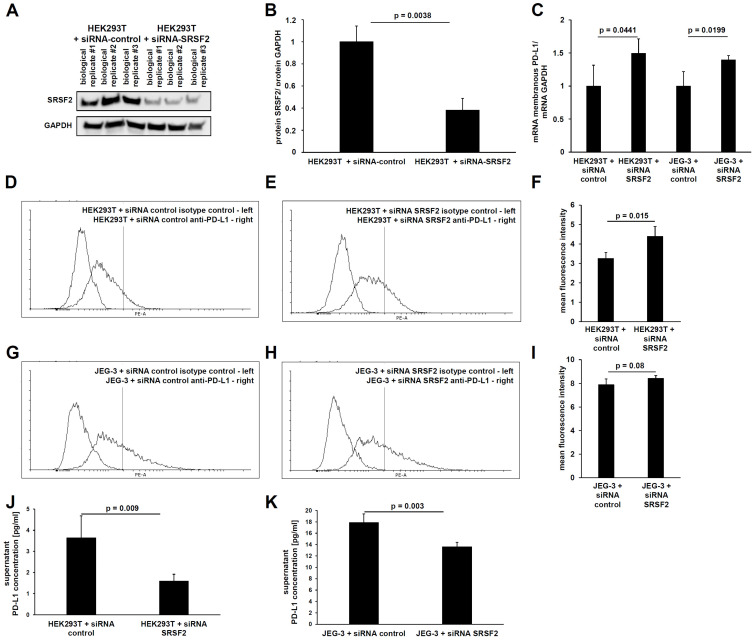
(**A**) Validation of siRNA-mediated SRSF2 knockdown after 72 h of transient transfection in HEK293T cells compared to the siRNA control by Western blot—including quantification of relative SRSF2 protein levels by pixel counting (**B**). Quantification of membranous full-length PD-L1 mRNA by qPCR in HEK293T and JEG-3 cells in combination with siRNA-mediated SRSF2 downregulation (**C**). Determination of the membranous PD-L1 protein in HEK293T cells (**D**,**E**) and JEG-3 cells (**G**,**H**) by flow cytometry after 72 h of transient transfection of the SRSF2 siRNAs or the siRNA control and determination of the mean fluorescent intensity for three biological replicates each (**F**,**I**). Measurement of secreted PD-L1 protein levels by ELISA in the corresponding cell culture supernatants after 72 h of transient transfection of SRSF2 siRNAs or siRNA control in HEK293T cells (**J**) and JEG-3 cells (**K**).

In analogy to SRSF2 overexpression, SRSF2 gene expression was also downregulated using siRNAs. For this purpose, the functionality of the siRNAs was first validated. This mechanism is based upon post-transcriptional gene regulation, which has the optimal readout at the protein level due to its predominant translational inhibition. Therefore, the siRNA-mediated downregulation of SRSF2 expression was analyzed by Western blot. Indeed, a statistically significant (*p* = 0.0038) reduction in SRSF2 protein was observed (Figure 3A,B).

To quantify the membrane-bound full-length PD-L1 mRNA splice variant using qPCR, primers and protocols based on the publication by Mahoney et al., 2019 [9], were used. This showed that both the HEK293T and JEG-3 transfectants still retained a statistically significant increase in the membranous full-length PD-L1 transcript due to siRNA-mediated SRSF2 downregulation (Figure 3C).

This was followed by the characterization of PD-L1 splicing. Due to the fact that siRNA transfection has a high transfection efficiency, even in difficult-to-transfect cell lines, the choriocarcinoma cell line JEG-3 was used in addition to the HEK293T cells. This cell line exhibits higher basal PD-L1 gene expression levels than HEK293T cells.

Subsequently, the protein content of the longer membrane-bound PD-L1 splice variant was measured using flow cytometry. This also revealed a statistically significant increase in membrane-bound PD-L1 protein due to the siRNA-mediated downregulation of the splice factor SRSF2 in HEK293T cells (*p* = 0.015) and a non-significant downregulation in JEG-3 cells (*p* = 0.08). The higher mean fluorescence intensity (MFI) clearly demonstrates the higher PD-L1 gene expression levels in JEG-3 cells compared to HEK293T cells (Figure 3D–I). Conversely, ELISA detected a clear and statistically significant reduction in the shorter secreted splice variant of PD-L1 in both HEK293T cells (*p* = 0.009) and JEG-3 cells (0.003) upon siRNA-mediated SRSF2 downregulation, whereby the ELISA data also revealed the higher basal PD-L1 gene expression of JEG-3 cells (Figure 3J,K).

For a more in-depth characterization of the splice factor SRSF2, its expression was examined using immunofluorescence in human tumor-free renal tissue. The tissue samples were taken by experienced pathologists from the renal cortex directly after nephrectomy. This showed that the splice factor SRSF2, as already known from the literature, could be detected predominantly within the nucleus. However, cytoplasmic localization of SRSF2 was also observed, which is quite possible, since translation generally occurs on the assembled ribosomes in the cytoplasm. Interestingly, the main SRSF2-positive cells were the epithelial cells of the renal tubular system as well as in the glomeruli. In all human kidney samples examined (*n* = 10; 5 male and 5 female), these structures were positive, but the percentage distribution of SRSF2-positive cells differed. In kidney samples from females, the mean values from ROIs of glomeruli, interstitium, and tubuli showed higher percentages of SRSF2 cells than was the case for males. However, this difference did not reach statistical significance. All the results together with the clinical parameters of the applied human kidney tissue samples are summarized in Table 1 and Table 2.

Additional tissue samples in which the physiological nature of PD-L1 expression can be regularly demonstrated are human placental trophoblasts and human tonsils, especially the lymphocytes of the germinal center (reactive center) [24].

**Figure 4:**
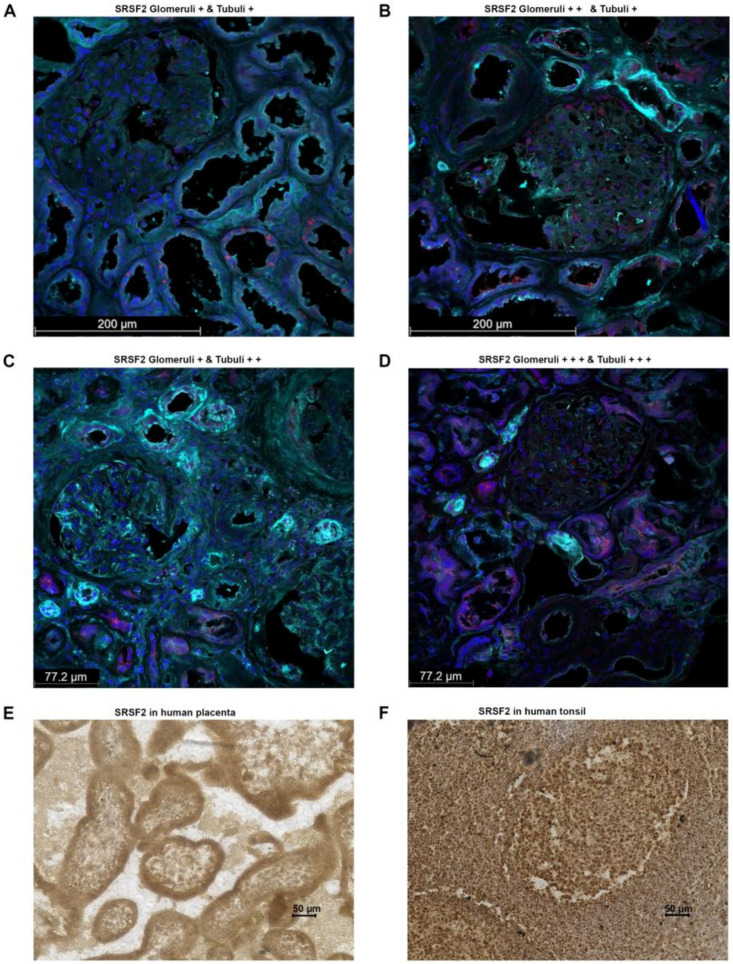
Characterization of SRSF2 protein in tumor-free human renal tissue specimens using immunofluorescence. Red: SRSF2; blue: nuclei; DAPI; turquoise WGA. The four selected examples illustrate the localization of SRSF2 mainly in the glomeruli and epithelial cells of the tubular system, as well as significant quantitative differences between different patient samples (**A**–**D**). Immunohistochemical staining of SRSF2 in human placenta (**E**) and tonsils (**F**).

Therefore, SRSF2 expression was analyzed in one human tissue sample each (placenta, tonsil) using immunohistochemistry. This showed that in the human placenta, SRSF2 expression is detectable in all cells, particularly nuclear and, to a lesser extent, cytoplasmic cells. However, trophoblasts exhibit a particularly high SRSF2 expression, suggesting the formation of the secreted PD-L1 splice variant in this tissue too, thereby contributing to the immunological tolerance, which is required for the fetus with its paternal antigens (Figure 4E). A similar picture is seen in the staining of the human tonsil. Here, out of all cells, the highest SRSF2 expression is observed in the nucleus and, to a lesser extent, within the cytoplasm. In particular, the B cells in the mantle zone show an even stronger SRSF2 expression than the T cells in the germinal center (Figure 4F).

The high SRSF2 expression in renal tubular epithelial cells is of great relevance in the context of pathophysiological PD-L1 expression. This includes monoclonal antibody-based treatments with anti-PD-L1 ICP inhibitors and therefore the relevant ratio between membranous and secreted PD-L1 splice variants, which can be observed with high frequency in renal cell carcinoma [25]. Interestingly, approximately 90% of renal tumors arise from the renal tubular epithelial cells, with the following most common subtypes: clear cell RCCs (ccRCCs; from epithelial cells of the proximal tubule), papillary RCCs (pRCCs; from the distal tubular epithelium), and chromophobe RCCs (chRCCs; from cells of the collecting duct) [25].

**Figure 5:**
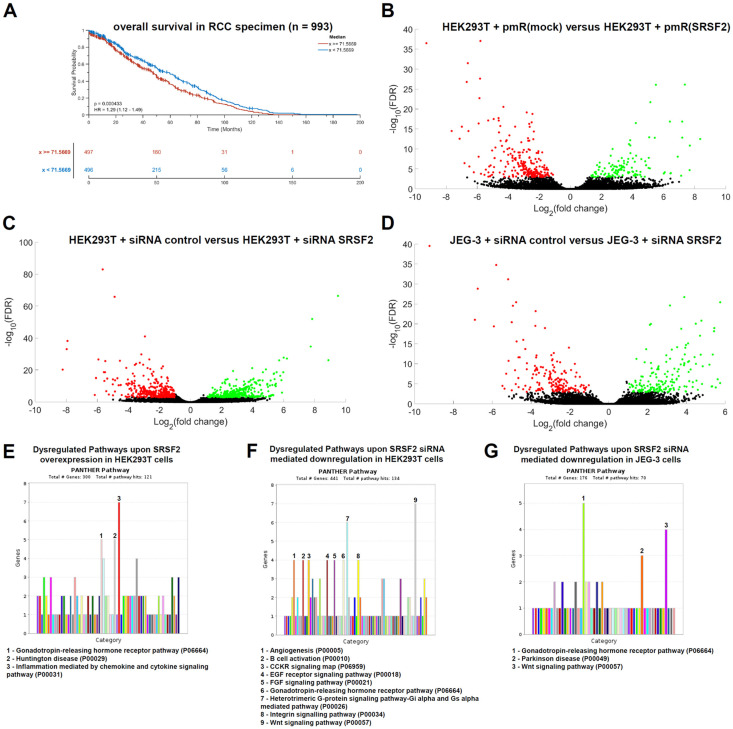
(**A**) In this Kaplan–Meier plot, the overall survival is analyzed in 993 RRC patients with respect to SRSF2 expression (low SRSF, blue curve, *n* = 496 versus high SRSF, red curve, *n* = 497). (**B**–**D**) Volcano plots show the dysregulated genes (red—statistically significantly reduced genes and green—statistically significantly induced genes) after RNA sequencing analyses of SRSF2 overexpression in HEK293T cells (**B**) and SRSF2 downregulation using siRNAs in HEK293T cells (**C**) and JEG-3 cells (**D**). Based on the respective RNA sequencing analyses, a functional analysis was performed using PANTHER v19 (https://pantherdb.org/webservices/go/overrep.jsp (accessed on 28 august 2025) [26]) to identify SRSF2-relevant cellular pathways (**E**–**G**).

Kaplan–Meier plots for RCC patients (including ccRCCs, pRCCs, and chRCCs) show that high SRSF2 expression (*n* = 497 specimens) is associated with a statistically significantly shorter overall survival than low SRSF2 expression (*n* = 496), with *p* = 0.0004 (Figure 5A).

In this data set, a possible correlation between SRSF2 gene expression with age and gender was also examined. While no statistically significant differences were observed with age (Spearman correlation coefficient = 0.0161; *p* = 0.611), a statistically significant gender effect was detected regarding SRSF2 gene expression (*p* = 0.0257), with females showing slightly higher SRSF2 gene expression than males in this data set.

To further analyze the deeper impact of the splice factor SRSF2 on tumor biology, we searched for additional transcripts that are dysregulated either by SRSF2 overexpression or by SRSF2 downregulation. Both directly and indirectly regulated putative target genes are of interest in this context. To address this question, we first performed RNA sequencing transcriptome analysis of HEK293T cells transfected with either the SRSF2 overexpression vector or the corresponding vector control (mock). For these analyses, cells were also harvested 72 h after transient transfection. In analogy, HEK293T and JEG-3 siRNA transfectants for SRSF2 downregulation were analyzed. The results are presented as volcano plots, with statistically significantly upregulated genes marked in green and downregulated genes marked in red (Figure 5B–D). Overexpression of SRSF2 resulted in a statistically significant increase of 91 transcripts and a downregulation of 187 transcripts (Supplemental Table S1).

A functional gene ontology (GO) enrichment analysis of the statistically significant dysregulated transcripts upon SRSF2 overexpression was performed. Therefore, the PANTHER online database was applied (https://pantherdb.org/webservices/go/overrep.jsp (accessed on 28 August 2025) [27]). SRSF2 overexpression caused statistically significant dysregulations within the gene expression of transcripts (upregulation *n* = 91; downregulation *n* = 187), which are functionally linked to the “Inflammation mediated by chemokine and cytokine signaling pathway” (*n* = 7), the “Huntington disease pathway” (*n* = 5), and the “Gonadotropin-releasing hormone receptor pathway” (*n* = 5) (Figure 5E). The siRNA-mediated downregulation of SRSF2 in HEK293T cells led to the statistically significant upregulation of 142 genes and the downregulation of 282 genes, of which most were functionally linked to the gonadotropin-releasing hormone receptor pathway (*n* = 9), the heterotrimeric G-protein signaling pathway/Gi alpha- and Gs alpha-mediated pathway (*n* = 6), and the Wnt signaling pathway (*n* = 10; Figure 5F,G). For validation, an analogous transcriptome analysis of siRNA-mediated SRSF2 downregulation in JEG-3 cells was performed. Statistically significantly upregulated (*n* = 96) and downregulated (*n* = 83) dysregulated genes were also identified. Functional GO enrichment analysis again identified the gonadotropin-releasing hormone receptor pathway, as well as Parkinson’s disease and the Wnt signaling pathway, as dysregulated genes (Figure 5G).

Thus, both SRSF2 overexpression and SRSF2 downregulation demonstrated that the splice factor SRSF2 particularly affects the gonadotropin-releasing hormone receptor pathway—a reproducible effect even in different cell lines. All statistically significantly dysregulated genes are summarized in Supplementary Table S1. These results are further characterized and put into context in Section 4.

## 4. Discussion

Following the success of monoclonal antibodies in the treatment of a variety of tumor diseases, current research is focused on identifying mABs to inhibit additional ICP axes besides the PD1-PD-L1 and CTLA4 axes and optimizing all of these therapies. One optimization strategy is combining them with chemotherapeutic agents or combining various ICP-mAbs to achieve additive effects. Additionally, efforts are being made to optimize the economical efficiency of these still relatively expensive immune therapies. Ondhia et al. [28] estimate the cost of atezolizumab therapy for advanced non-small-cell lung cancer (NSCLC) in Canada at approximately USD 130,563, compared to USD 45,490 for docetaxel therapy. Meanwhile, for RCC patients, the following cost is given in detail in the literature: avelumab plus axitinib—USD 174,725 annually [29].

Such diverse optimization considerations should include a prior analysis of each tumor tissue with regard to the expression of the target protein of the respective putative therapeutic mAb. This refers not only to the protein level per se, but qualitative analyses should also be carried out in this regard—including the characterization of the presence of secreted splice variants or, even better, a direct characterization of the ratio between membranous and secreted PD-L1. However, this diagnostic procedure must not be financially prohibitive and must be easy to perform for the laboratories involved, so that the identification of relevant splice factors as a simple additional IHC marker represents a logical step in anti-tumor immunological research.

The present data demonstrate that the splicing factor SRSF2 exhibits an association with PD-L1 splicing, which possibly has clinical relevance for corresponding ICP therapies based on mAbs against PD-L1. For clinical relevance, it is completely irrelevant whether this regulation is direct, indirect, and/or both, which could be analyzed through appropriate mutagenesis experiments for the putative SRSF2 binding site at the corresponding exon/intron boundary or through cross-linking immunoprecipitation assays. Looking ahead, the possible relevance of this interaction with immune effector cells for the SRSF2-induced shift in PD-L1 alternative splicing towards increased formation of secreted PD-L1 splice variants could be further characterized by respective in vitro T cell lysis assays, using T killer cells that can also be combined with the corresponding HLA-restricted SRSF2 transfectants (SRSF2 upregulation/downregulation) as putative target cells.

The SRSF2 gene is located in the human genome on the long arm of chromosome 17—with the exact position being 17q25.1. SRSF2 is a serine-/arginine-rich splicing factor (SRSF). In humans, there are twelve such RNA-binding proteins known, which regulate splice site recognition and spliceosome assembly during precursor messenger RNA splicing; in particular, SRSFs are important trans-acting factors that regulate nearly every step of alternative splicing, whereby SRSFs facilitate the definition of exon–intron boundaries [30].

According to the Human Protein Atlas, the SRSF2 protein can primarily be detected in the nucleoplasm and, to a lesser extent, in the cytoplasm [31]. This was actually the case in the analyzed renal tissues. However, strong differences were observed regarding the protein levels in the respective kidney specimen as well as in the sublocalization, for example, between the glomeruli and the tubuli.

Another approach to identify further direct SRSF2 target mRNAs is through cross-linking and immunoprecipitation (CLIP) experiments. However, the overall impact of SRSF2 on the transcriptome should be analyzed—including direct and indirect upregulated and downregulated target genes. We aimed to obtain insights into dysregulated pathways for a deeper understanding of the strong statistically significant negative correlation between SRSF2 expression and overall survival.

Twelve different SRSFs have now been described in the literature, most of which contain one or even two N-terminal RNA recognition motifs (RRMs) and a C-terminal RS domain, except for SRSF7, which contains an additional zinc-binding domain in the middle [30]. At this point, the different affinities of these binding sites could result in artifacts in the CLIP results and obscure a global understanding of SRSF2 in the cellular transcriptome. Thus, it is conceivable that SRSF2 binds to certain target mRNAs with one or the other RRM, and then, during immunoprecipitation, these transcripts are enriched and identified, but, for example, very low-expressed transcripts of potentially regulatory but highly relevant genes are simply lost during immunoprecipitation. Furthermore, transcripts bound to the other RRM with low affinity could potentially be lost during CLIP, while high-affinity transcripts bound to the other RRM remain intact, which helps to quantify artifactual results.

This analysis is especially relevant given that SRSF1 has already been characterized as an oncoprotein in tumors [32], making it even more important to analyze the full impact of SRSF2 on the cellular transcriptome—independent of the direct or indirect regulation of SRSF2 overexpression/downregulation. In addition to siRNAs, it is also possible to inhibit SRSF activity using various inhibitors, including different indole derivatives. One study identified C77 and C83 as specific SRSF2 inhibitors [33].

In tumor diseases, SRSF2 generally appears to be involved in the process of malignant transformation, e.g., in hepatocellular carcinoma [34]. Furthermore, SRSF2 overexpression and its direct involvement in tumor progression have been observed in lung tumors [35]. Even in hematopoietic tumor diseases such as myelodysplastic syndromes and acute myeloid leukemia, an association with SRSF2 mutations has been observed, e.g., in correlation with malignant transformation, reduced overall survival, and increased therapy resistance [36]. Our transcriptome analyses using SRSF2 overexpression and siRNA-mediated SRSF2 knockdown revealed the gonadotropin-releasing hormone receptor pathway and the Wnt signaling pathway as dysregulated pathways. Dysregulation of the first pathway could affect cell cycle regulation and proliferation, and its dysregulation is usually observed in several human malignant tumors of the urogenital tract, including cancers of the endometrium, ovary, urinary bladder, and prostate [37]. WNT pathway dysregulation could additionally contribute to direct malignant transformation and tumor progression, and this pathway is also known to be dysregulated in urological tumor diseases [38].

**Figure 6:**
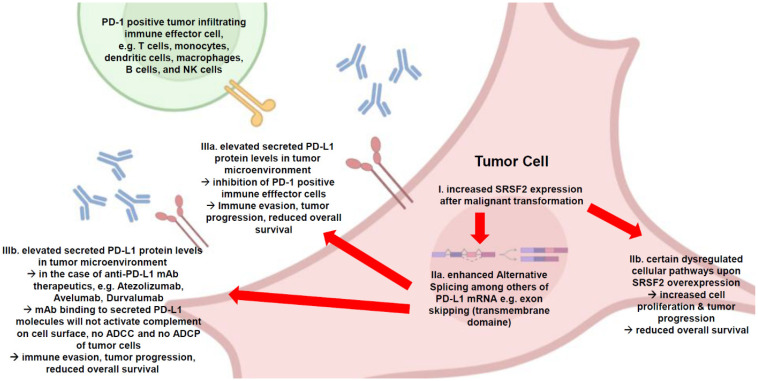
This schematic overview shows the consequences of increased SRSF2 expression after malignant transformation in a tumor cell, using the example of enhanced PD-L1 alternative splicing. This results in the increased formation of the secreted PD-L1 splice variant and its release into the tumor microenvironment for effective inhibition of immune effector cells, even at a distance from the actual tumor cell. The implications for the therapeutic use of mAbs against PD-L1 are illustrated. This antibody binding away from the target cell means that the tumor cell is not threatened by complement activation with membrane attacking complex, ADCC, or even ADCP, which could result in reduced efficacy of these expensive therapies. Therefore, careful attention must be paid to the prior selection of putative mAbs regarding the potential relevance of SRSF2 as a putative diagnostic factor (created with BioRender.com).

The altered ratio of membrane-bound to secreted PD-L1 could play a decisive role in the reduced overall survival observed in many tumor diseases with elevated SRSF2 expression—regardless of whether a therapy with mAbs against the PD-1/PD-L1 ICP axis is applied, because it also involves the inhibition of tumor-infiltrating immune effector cells. Furthermore, an increased concentration of secreted PD-L1 in the tumor microenvironment could inhibit immune effectors efficiently even before they reach the tumor cells. These relationships are summarized in a schematic overview (Figure 6).

## 5. Conclusions

The therapeutic success of mAbs against tumor ICP axes continues. With the increasing awareness of the possibilities for additive treatments with combinations of different mAbs, the need to optimize these expensive therapies is increasing.

At the same time, evidence is accumulating that altered alternative splicing occurs in tissues after malignant transformation [39], which can be explained by altered gene expression of the splicing factors involved, with the splicing factor SRSF2 already described in the literature as being relevant to this process [4].

The most important ICP axis is probably the PD-1/PD-L1 axis, in particular PD-L1, which, as an immunomodulatory molecule, can be pathophysiologically detected at high frequencies in various tumor diseases after malignant transformation. In this study, we show that the splicing factor SRSF2 is associated with the alternative splicing of PD-L1, leading to increased formation of the secreted PD-L1 splice variant.

This finding is crucial, as it will likely negatively affect the efficacy of expensive ICP therapies with mAbs explicitly targeting PD-L1 (e.g., atezolizumab, avelumab, and durvalumab). Therefore, tumor SRSF2 expression levels should be considered when selecting appropriate mAbs. In the case of elevated SRSF2 gene expression, mAbs targeting PD-1 (e.g., nivolumab, pembrolizumab) may be a better alternative, or even a switch to anti-CTLA4 mAbs (e.g., ipilimumab, tremelimumab) or other ICP axes, e.g., TIGIT (vibostolimab, tiragolumab) or ILT2 (BND-22 [4]).

Indeed, the combination of multiple mAbs (nivolumab plus ipilimumab) is superior to the use of a single mAb (nivolumab) and sunnitinib in terms of overall survival rate in advanced renal cell carcinoma; 75% versus 60% [40]. However, recent studies show that modern tyrosine kinase inhibitors (TKIs) such as cabozantinib are significantly superior to older TKIs such as sunitinib. In terms of progression-free survival, the following results were obtained: 16.4 versus 8.3 months [41].

Following our hypothesis, improvements could potentially be achieved for almost all mAb-based therapies because, as published [4], PD-1, CTLA-4, HLA-G, and many other ICP molecules also undergo alternative splicing with the creation of soluble isoforms in full analogy to our findings presented in this study. It should also be noted that TKIs have significant side effects, such as cardiotoxicity (particularly the induction of hypertension), so optimized antibody therapies will certainly dominate in the future, provided costs are minimized, e.g., through better molecular biological characterization of the patient in favor of optimized individualized therapy.

The presence of other ICP axes and the possibly associated mAb-based inhibition of these other putative axes in the form of mAb combination therapies will also necessitate (i) better molecular biological characterization of the respective tumor and (ii) cost reduction for mAb therapies, possibly due to dose reduction enabled by optimal molecular biological diagnostics in advance.

## Figures and Tables

**Table 1:** Tabular summary of the most important clinical parameters of the patient samples used for immunofluorescence and the scoring of the SRSF2 protein level.

Kidney Tissue Specimen	Age [Years] at Nephrectomy	Male/ Female	Diagnosis/Reason for Nephrectomy
#1	80	female	urothelial carcinoma
#2	86	female	urothelial carcinoma
#3	51	male	renal cell carcinoma
#4	56	male	non-functional kidney
#5	47	female	recurrent pyelonephritis, continuous PNS care
#6	72	female	renal cell carcinoma
#7	67	male	renal cell carcinoma
#8	73	male	urothelial carcinoma
#9	83	male	urothelial carcinoma
#10	79	female	renal cell carcinoma

**Table 2:** Percentage distribution of SRSF2-positive cells in renal tissue samples broken down by the three regions of interest using QuPath: glomeruli only, complete interstitium, and tubuli only [21].

Female kidney tissue specimen	Percentage of SRSF2-positive cells—glomeruli only (females)	Percentage of SRSF2-positive cells—glomeruli only (males)	Male kidney tissue specimen
#1	81	32	# 3
#2	19	9	#4
#5	77	14	#7
#6	35	12	#8
#10	11	54	#9
Mean	44.5	24.1	
Standard deviation	32.3	19	
*p* (two-sided)	0.26		
Female kidney tissue specimen	Percentage of SRSF2-positive cells—complete interstitium (females)	Percentage of SRSF2-positive cells—complete interstitium (males)	Male kidney tissue specimen
#1	83	57	#3
#2	31	24	#4
#5	91	26	#7
#6	41	45	#8
#10	51	31	#9
Mean	59.5	36.5	
Standard deviation	26.3	14.3	
*p* (two-sided)	0.12		
Female kidney tissue specimen	Percentage of SRSF2-positive cells—tubuli only (females)	Percentage of SRSF2-positive cells—tubuli only (males)	Male kidney tissue specimen
#1	92	76	#3
#2	95	60	#4
#5	99	60	#7
#6	97	80	#8
#10	23	64	#9
Mean	81.1	67.9	
Standard deviation	32.6	9.3	
*p* (two-sided)	0.41		

## Data Availability

All data used are provided in this manuscript or can be found in the Supplementary Material. Further information may be made available by the corresponding author at simon.jasinski-bergner@mhb-fontane.de.

## Supplementary Materials

The following supporting information can be downloaded at https://www.mdpi.com/article/10.3390/cancers17213463/s1, Table S1: Statistical significant upregulated genes.
